# Magnetically Hollow Pt Nanocages with Ultrathin Walls as a Highly Integrated Nanoreactor for Catalytic Transfer Hydrogenation Reaction

**DOI:** 10.1002/advs.201802132

**Published:** 2019-02-07

**Authors:** Yongjian Ai, Zenan Hu, Lei Liu, Junjie Zhou, Yang Long, Jifan Li, Mingyu Ding, Hong‐Bin Sun, Qionglin Liang

**Affiliations:** ^1^ Key Laboratory of Bioorganic Phosphorus Chemistry & Chemical Biology (Ministry of Education) Beijing Key Lab of Microanalytical Methods & Instrumentation Department of Chemistry Center for Synthetic and Systems Biology Tsinghua University Beijing 100084 P. R. China; ^2^ Department of Chemistry Northeastern University Shenyang 110819 P. R. China

**Keywords:** amines, catalytic transfer hydrogenation, integrated nanoreactors, Pt nanocages, ultrathin walls

## Abstract

Fabricating efficient and stable nanocatalysts for chemoselective hydrogenation of nitroaromatics is highly desirable because the amines hold tremendous promise for the synthesis of nitrogen containing chemicals. Here, a highly reactive and stable porous carbon nitride encapsulated magnetically hollow platinum nanocage is developed with subnanometer thick walls (Fe_3_O_4_@*sn*Pt@PCN) for this transformation. This well‐controlled nanoreactor is prepared via the following procedures: the preparation of core template, the deposition of platinum nanocage with subnanometer thick walls, oxidative etching, and calcination. This highly integrated catalyst demonstrates excellent performance for the catalytic transfer hydrogenation of various nitroaromatics and the reaction can reach >99% conversion and >99% selectivity. With the ultrathin wall structure, the atom utilization of platinum atoms is highly efficient. The X‐ray photoelectron spectroscopy results indicate that partial electrons transfer from the iron oxides to Pt nanowalls, and this increases the electron density of *sn*Pt nanoparticles, thus promoting the catalytic activity for the transfer hydrogenation of nitroaromatics. For the reduction of 4‐nitrophenol, the reaction rate constant *K*_app_ is 0.23 min^−1^ and the turnover frequency (TOF) is up to 3062 h^−1^. Additional reaction results illustrate that this magnetic nanoreactor can be reused more than eight times and it is a promising catalytic nanoplatform in heterogeneous catalysis.

## 1 Introduction

Fabricating stable and high catalytic activity nanocatalyst is an eternal pursuit for catalysis and material scientists, not only for their fundamental scientific interest, but also for many industry technological applications.^1^, ^2^, ^3^, ^4^, ^5^, ^6^, ^7^, ^8^, ^9^ The precious metals (such as Au, Ag, Ru, Rh, Pd, and Pt) are indispensable component of many heterogeneous catalysts, which are used in chemical, pharmaceutical, petroleum, and energy industry. These catalysts usually own unique properties, such as excellent electronic conduction, numerous of reactive corners, and high‐specific surface area.^10^, ^11^, ^12^, ^13^, ^14^, ^15^ More than atomic scale size, the ultrasmall noble metal nanoparticles (NPs) especially at subnanometer level possess unprecedented properties compared to conventional bulk materials. The exploitation of novel subnanometer materials for heterogeneous catalysis is one of frontier research.^16^, ^17^, ^18^, ^19^, ^20^, ^21^, ^22^

High exposure rate of noble metal atom is necessary for a practical catalyst due to the expense of noble metal.^23^, ^24^, ^25^, ^26^, ^27^ Reducing the particle size is an instant method; however, the solid nanoparticles tend to reunite irreversibly during catalytic reactions procedure due to the high surface energy. This generally leads to a dramatic loss of the original catalytic activity.^28^, ^29^, ^30^ What's more, as the catalyst nanoparticles are very small, when the catalyst is separated from catalytic reaction system for further reuse, the cumbersome filtration protocol usually brings a bunch of headaches.^31^, ^32^, ^33^, ^34^, ^35^, ^36^, ^37^ It has been reported that the activity site of the catalyst was the surface atoms (about three atomic layers thick), while the extra atoms were useless for the catalytic system.^38^, ^39^ Considering the tricky problems for application of noble metals, the fabrication of stable subnanometer structures that enhance the atom utilization efficiency and reproducibility is highly significant both for academic research and industrial applications.^40^, ^41^, ^42^ Fortunately, the rapid development of nanotechnology and nanoscience has encouraged material scientists and chemists to fabricate molecular level designed nanomaterials to a great degree.^43^ Many pioneers' works have been reported on dispersing the noble metal NPs on the commonly oxide supports and to construct highly efficient catalyst. However, enhancement of the noble metal loading efficiency for a heterogeneous catalyst is still a great challenge.^44^, ^45^, ^46^

Herein, we describe the synthesis of porous carbon nitride encapsulated magnetic hollow platinum nanocubes with a subnanometer thickness wall (Fe_3_O_4_@*sn*Pt@PCN). The nanocatalyst was fabricated through the preparation of core template, deposition of subnanometer thick platinum nanocage, oxidative etching, and calcination protocols, sequentially. Catalytic tests validated that the magnetically platinum nanoreactor displayed excellent catalytic performance in the catalytic transfer hydrogenation of nitroaromatics with NaBH_4_. The electron exchange between iron oxides and Pt nanowall accelerates the electron transfer during the catalytic transfer hydrogenation reaction and the synergistic effect improves the catalytic activity. The platinum wall was applied as active sites for the catalytic reactions with high atom utilization efficiency. As this catalyst exists as a nanocage structure with a magnetic kernel and further encapsulated by carbon nitride, the catalyst can be easily recovered and reused for more than eight times without obvious activity loss.

## 2 Results and Discussion

**Figure 1:**
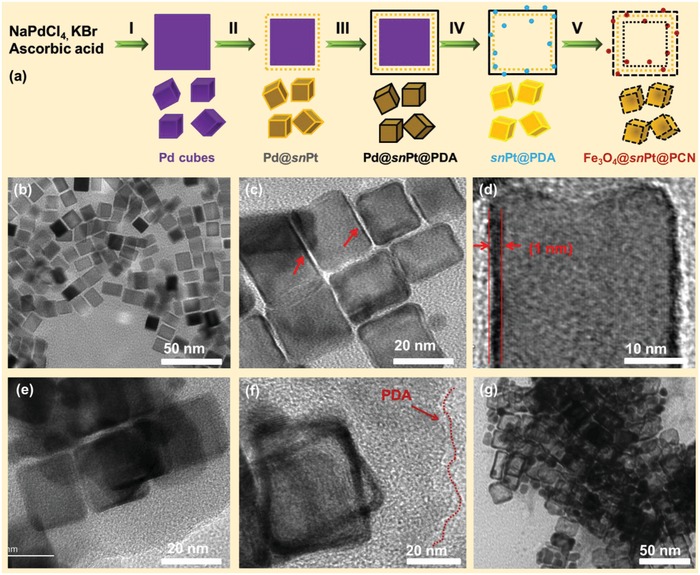
a) Schematic illustration for the fabrication of Fe_3_O_4_@*sn*Pt@PCN: (I) fabrication of Pd nanocubes, (II) fabrication of Pd@*sn*Pt, (III) fabrication of Pd@*sn*Pt@PDA, (IV) oxidative etching of Pd@*sn*Pt@PDA by FeCl_3_ and HCl solution, and (V) fabrication of Fe_3_O_4_@*sn*Pt@PCN. HR‐TEM images of the catalyst in each stage: b) Pd template nanocubes, c,d) Pd@*sn*Pt, e) Pd@*sn*Pt@PDA, f) *sn*Pt@PDA, and g) Fe_3_O_4_@*sn*Pt@PCN.

The detailed formation of the Fe_3_O_4_@*sn*Pt@PCN was illustrated in **Figure** **1**a. First, the NaPdCl_4_ was used to fabricate the Pd nanocube, which was used as a template in the following. Then, the Pt was carefully deposited to generate the subnanometer wall. After that, the Pd@Pt was encapsulated by the polydopamine (PDA) then the Pd core was etched away through the oxidative etching method with HCl and FeCl_3_. Finally, the residue was calcined by the tube furnace in N_2_ atmosphere. The morphologies of intermediates for each stage were well characterized by high‐resolution transmission electron microscopy (HR‐TEM) and its accessories apparatus. Figure 1b and Figure S1 in the Supporting Information show the Pd template nanocubes are highly uniform dispersed with the size of 20 nm. Furthermore, the X‐ray photoelectron spectroscopy (XPS) (Figure S2, Supporting Information) characterization demonstrates that the Pd nanocubes are successfully fabricated with high purity. Figure 1c,d and the scanning transmission electron microscopy (STEM) in Figure S3 in the Supporting Information obviously demonstrate that the Pt atom is deposited on the surface of Pd nanocubes, and the thickness of the wall is subnanometer level. What's more, the selected area electron diffraction (SAED), energy‐dispersive spectrometer (EDS) mapping, and XPS characterizations in Figures S3–S5 in the Supporting Information clearly confirm that the Pd@*sn*Pt is successfully fabricated as the Figure 1a designed. The result of the Figure 1e illustrates that the Pd@*sn*Pt is encapsulated by the PDA and the thickness of the PDA layer was about 15 nm. The STEM and EDS mapping in Figure S6 in the Supporting Information highly consent to the HR‐TEM result. The linear scan (Figure S7, Supporting Information) further verified that the Pd@*sn*Pt@PDA was synthesized smoothly. The Figure 1f and Figure S8 in the Supporting Information show that the Pd template at the center sites of the Pd@*sn*Pt@PDA has been etched away by the oxidative processing with the FeCl_3_ and HCl solution to obtain the hollow *sn*Pt@PDA, where the Fe^3+^ is chelated in the structure. Finally, the *sn*Pt@PDA structure was annealed at 500 °C for 3 h in the high purity N_2_ atmosphere with a heating rate of 5 °C min^−1^. Finally, we obtain the Fe_3_O_4_@*sn*Pt@PCN yolk‐shelled nanocubes catalyst (Figure 1g).

**Figure 2:**
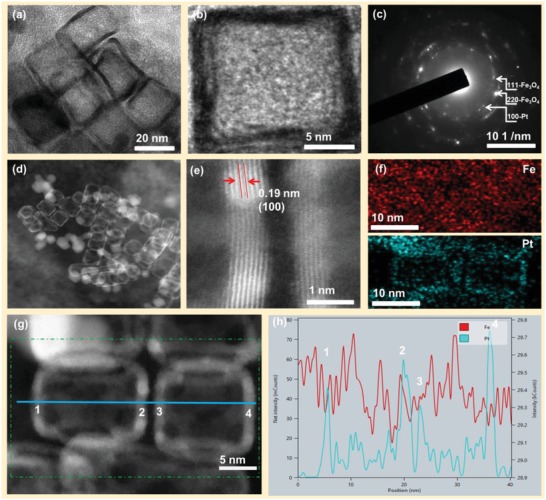
a,b) HR‐TEM images, c) SAED pattern, d) HAADF‐STEM image, e) high‐magnification HAADF‐STEM image, f) EDS mappings, and g,h) line scanning of the Fe_3_O_4_@*sn*Pt@PCN nanocatalyst.

Furthermore, detailed characterization of the fabricated Fe_3_O_4_@*sn*Pt@PCN nanocatalyst was conducted. The HR‐TEM images of the Fe_3_O_4_@*sn*Pt@PCN in the **Figure** **2**a,b clearly illustrate that the hollow *sn*Pt@PCN was fabricated and the wall of the Pt nanocage was about 1 nm, which contains about six atomic layers of Pt. The SAED pattern (Figure 2c) shows the characteristic diffraction rings that are corresponding to the Pt (100), Fe_3_O_4_ (111), and (220), respectively. The field‐emission aberration‐corrected transmission electron microscope was further used to characterize the Fe_3_O_4_@*sn*Pt@PCN. The high‐angle annular dark field (HAADF)‐STEM images (Figure 2d,e and Figure S9, Supporting Information) show that the nanoreactor has been fabricated as we designed. Furthermore, the *d*‐spacing of adjacent fringes observed by the high HAADF‐STEM image is 0.19 nm, which is attributed to the (100) crystal plane of Pt. The EDS mapping (Figure 2f) of Pt and Fe demonstrated that the Fe_3_O_4_ was distributed in the Pt nanocage. The result of line scanning (Figure 2g,h) of the nanocages from 1 to 4 was confirmed. Following the above characterization, for this catalyst, the Fe_3_O_4_ was distributed in the hollow *sn*Pt and PCN. What's more, some Fe_3_O_4_ may replace the Pt atoms of the subnanometer walls. The inductively coupled plasma mass spectrometry (ICP‐MS) characterization demonstrates that the content of the Pt is 1.91 wt%. The X‐ray diffraction (XRD) characterization of the Fe_3_O_4_@*sn*Pt@PCN catalyst was showed in Figure S10 in the Supporting Information, it is clearly observed that the intense diffraction peaks of 2θ = 31.1°, 35.5°, 43.3°, 53.5°, 57.1°, and 63.2° are corresponded to the (220), (311), (400), (511), and (440) lattice facets of the Fe_3_O_4_ (JCPDS: 00‐003‐0862), respectively. Obviously, no characteristic diffraction peak correspond to Pt was observed in the XRD patterns, this may because the loading of Pt was low and the subnano size of the Pt walls did not promote in the XRD. Furthermore, with the oxidative etching treatment, the Pd nanocubes were etched away and the corresponding peaks disappeared.

**Figure 3:**
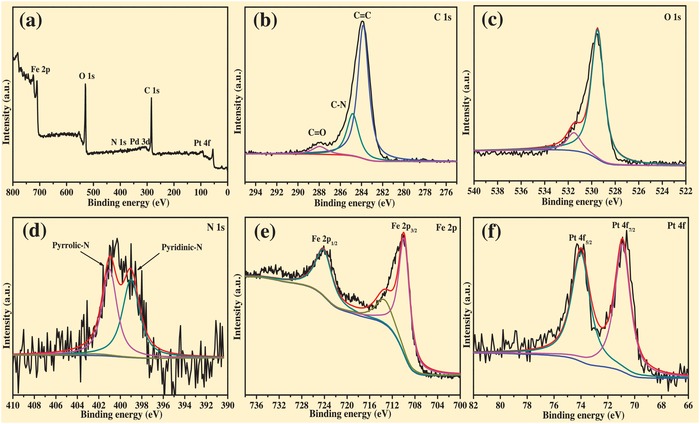
XPS characterization of the magnetically hollow Fe_3_O_4_@*sn*Pt@PCN nanocatalyst a) survey spectra, b) C 1s spectra, c) O 1s spectra, d) N 1s spectra, e) Fe 2p spectra, and f) Pt 4f spectra.

The XPS characterization results (**Figure** **3**) demonstrate that the magnetic hollow Fe_3_O_4_@*sn*Pt@PCN nanocatalyst was mainly composed with five elements, namely, Fe, C, O, N, and Pt according to the survey XPS spectra. The XPS spectrum of C 1s shows that the binding energies of 283.5, 285.4, and 288.1 eV are corresponding to the graphitized carbon, carbon nitride, and carbonyl, respectively (Figure 3b,c). According to the N 1s XPS spectra (Figure 3d), the nitrogen exists as pyridine‐like nitrogen (398.43 eV) and pyrrole‐like nitrogen (401.68 eV), respectively. From the Figure 3d, the obvious peaks at 710.52 and 724.83 eV are attributed to Fe 2p_3/2_ and Fe 2p_1/2_ of Fe_3_O_4_, respectively. The XPS spectra of the Pt 4f show that the binding energy of the Pt 4f_7/2_ and Pt 4f_5/2_ at 74.23 and 70.34 eV reveals the platinum was zerovalent Pt (Figure 3e). Figure S10 in the Supporting Information further confirms that the Pd in the nanocatalyst was successfully etched through the oxidation etching protocol.

**Figure 4:**
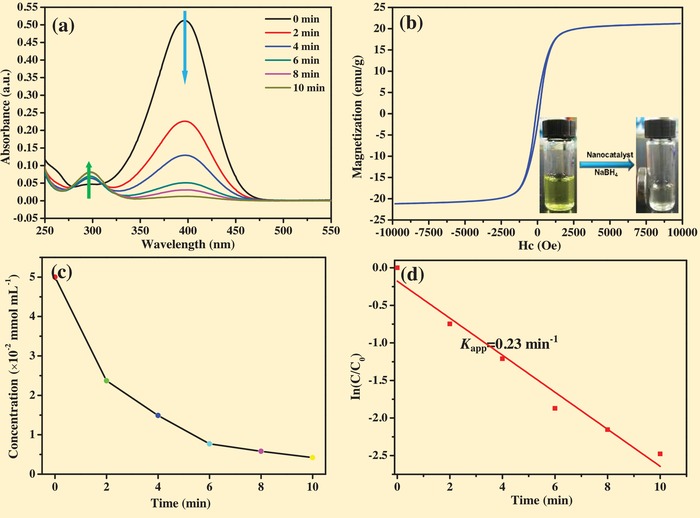
a) The UV–vis absorption spectra for the catalytic hydrogenation of 4‐NP over the highly active Fe_3_O_4_@*sn*Pt@PCN nanocatalyst. b) Room‐temperature hysteresis loops of Fe_3_O_4_@*sn*Pt@PCN (inset one was the typical optical image of reaction solution and the catalyst separated by an additional magnet). c) The concentrations of 4‐NP as a function of time in the presence of the Fe_3_O_4_@*sn*Pt@PCN catalyst. d) Plot of ln(*C*/*C*_o_) versus time.

We applied this nanoreactor in the catalytic transfer hydrogenation of nitroaromatics to afford the aromatic amines. Due to the limited availability of natural products, the large amount demand of the dyestuffs, pharmaceutical, agricultural chemicals, and polymers for aromatic amines was satisfied by the catalytic transfer hydrogenation of their corresponding nitroarenes.^47^ It was reported that the requirement of anilines compounds is more than 4 million tons per year and the value of these chemical intermediates will increase to £10.17 billion by 2020.^48^, ^49^ Many previous works have reported about this transformation over noble metal and noble metal‐free catalyst. Noble metal catalysts have attracted much more attention owing to the advantages of high activity and high selectivity; therefore, developing novel noble metal catalyst for this transformation is a hot topic.^50^, ^51^ In recent years, we have focused on this highly important reaction and have fabricated a series of catalysts for this transformation.^52^, ^53^, ^54^, ^55^, ^56^, ^57^, ^58^ For the Fe_3_O_4_@*sn*Pt@PCN, we first conducted the catalytic activity test experiment through the catalytic transfer hydrogenation of 4‐nitrophenol (4‐NP), which is a representative nitro compound for this protocol. The conversion and chemoselectivity of reaction were applied to evaluate the optimization, and the experiment results concluded in **Table** **1** were detected by UV–vis spectroscopy, thin layer chromatography (TLC), and gas chromatography‐mass spectrometer (GC‐MS). It is obvious that without the catalyst this reaction cannot execute. The Pd nanocube can successfully catalyze the reduction of 4‐NP in 20 min (entries 1–2). The Pd@*sn*Pt mediated experiment demonstrated that the reduction of 4‐NP to the corresponding 4‐AP was completed in 15 min (entry 3). As the catalyst was encapsulated by the PDA, the catalytic activity became weaker and it took 35 min for the complete reduction of 4‐NP (entry 4). What's more, neither the PDA nor porous carbon nitride (PCN) (fabricated by the carbonization of the PDA) showed catalytic activity for this reaction (entries 5–6). The UV–vis spectroscopy (**Figure** **4**a) demonstrated that the absorption peak of 4‐NP (about 400 nm) was gradually turned to low intensity, at the same time the peak of 4‐AP (about 300 nm) was gradually increased. This phenomenon indicated that the 4‐NP was successfully transformed to the 4‐AP in 10 min (entries 7–8) over the Fe_3_O_4_@*sn*Pt@PCN.

**Table 1:**
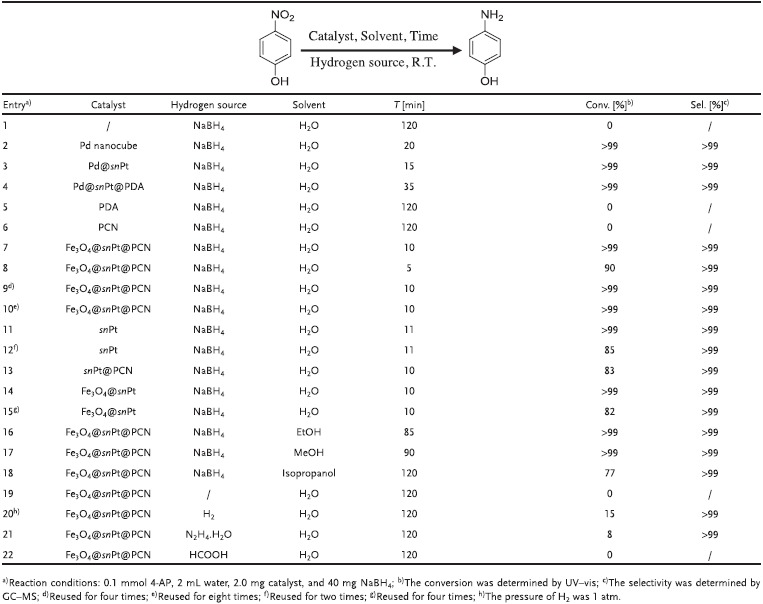
Optimization of reaction parameters for the catalytic transfer hydrogenation of 4‐nitrophenol

**Figure 5:**
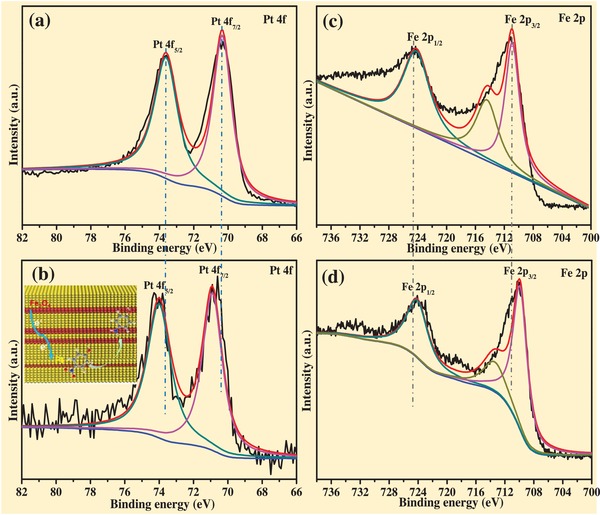
XPS profiles of Pt 4f in a) Pd@*sn*Pt, b) Fe_3_O_4_@*sn*Pt@PCN; Fe 2p in c) Fe_3_O_4_, and d) Fe_3_O_4_@*sn*Pt@PCN.

The reaction photograph showed that the yellow reaction solution was converted into the colorless one, which means the 4‐NP was successfully reduced to the corresponding 4‐AP. The vibrating sample magnetometer characterization (Figure 4b) of the Fe_3_O_4_@*sn*Pt@PCN demonstrated that the saturation magnetization (Ms) value was about 20 emu g^−1^, and this meant that the catalyst was magnetically separable. As the inset image of Figure 4b showed, an external magnet can separate the catalyst from the reaction solution without trivial work and the catalyst can be applied for further reuse. The concentrations of 4‐AP and 4‐NP versus reaction time indicated that the selectivity toward 4‐AP was close to 100%. As the NaBH_4_ is excess for this reaction system, the reaction can be considered as a pseudo‐first‐order reaction, and the pseudo‐first‐order rate constants (*k*_app_) were calculated from plots of ln(*C*/*C*_0_) versus time (*t*). Figure 4d shows the plot of ln(*C*/*C*_0_) versus time (*t*) for the Fe_3_O_4_@*sn*Pt@PCN catalyst system, and the good linear relationship confirms the assumption of pseudo‐first‐order kinetics. The rate constant for the 4‐NP reduction determined from the slope of the plot was 0.23 min^−1^. In addition to the catalytic activity, the stability and recyclability are significant to evaluate a novel catalyst system. As shown in the entries 9–10, the conversion and selectivity did not exhibit decrease even the Fe_3_O_4_@*sn*Pt@PCN was reused for more than eight times. Combined with the HR‐TEM characterization result in Figure S11 in the Supporting Information, it was demonstrated that this catalyst was highly active, stable, and recyclable. The *sn*Pt demonstrated the analogous catalytic activity to the Fe_3_O_4_@*sn*Pt@PCN; however, the catalyst cannot be reused for more than two times (entries 11–12) and the cumbersome centrifugal protocol was required to separate and enrich the *sn*Pt catalyst. The PCN encapsulated *sn*Pt catalyst showed less catalytic activity than the Fe_3_O_4_@*sn*Pt@PCN, and it can only convert 83% 4‐NP to anilines in 10 min (entry 13). The Fe_3_O_4_@*sn*Pt catalyst exhibited the excellent catalyst for this reaction, the catalytic activity decreased (entries 14–15) after four cycles. As we used other solvents for this catalytic system, such as EtOH, MeOH, and isopropanol, the catalytic activity was not satisfied (entries 16–18). The hydrogen sources were also explored and the results showed that other reductants were not much suitable for this catalytic system (entries 19–22). As we compared the entry 8 and entry 14, we noted that introducing Fe_3_O_4_ to the catalyst did not only make this catalyst easily separable by an extra magnetic field, but also improved the catalyst's activity. Further, XPS characterization was conducted to explain this phenomenon. The XPS measurements revealed that a partial electron transferred from the Fe_3_O_4_ nanoparticles to the *sn*Pt. As shown in **Figure** **5**, the binding energy of the Pt 4f_5/2_ increased from 73.81 to 74.23 eV and the counterpart of the Fe_3_O_4_ was decreased from 724.18 to 724.02 eV. This suggests that electron density of *sn*Pt nanoparticles was increased in Fe_3_O_4_@*sn*Pt@PCN, which further increased the hydrogenation activity.^5^ Further reasons that contribute to increase the catalytic activity and stability of the Fe_3_O_4_@*sn*Pt@PCN could be attributed to the following: the ultrathin platinum layer was completely exploited as active site and the atom utilization efficiency was up to 100%. The activity sites Fe_3_O_4_@*sn*Pt are encapsulated by the PCN, which has good mass transport and provides sinter‐resistance that prevents the migration and agglomeration of catalyst center during the catalytic reactions. Furthermore, the nitrogen atom of PCN further stabilized the metal atoms. Thus, the above reasons synergistically ensure the catalyst system and lead the catalyst maintains its initial activity without any deactivation after reused for eight recycles.

The applied scope is a significant metrical to evaluate a new catalytic system. In order to certify the catalytic activity and chemoselectivity of the Fe_3_O_4_@*sn*Pt@PCN nanocatalyst that we recently developed, we attempted the catalytic hydrogenation of nitroarenes with different functional groups. As shown in **Table** **2**, all substrates completed the reactions within 10 min and the reaction results were satisfactory. In addition, in all cases, the azoxy, azo, and hydrazo compounds, which were usually generated as by‐products in the catalytic hydrogen transfer nitroarenes, were not detected. For the simple nitrobenzene, 4‐nitrophenol and 4‐nitrotoluene, the reaction was successfully constructed (entries 1–3). Although the halogenated aromatic amines were significant intermediates for the synthesis of fine chemicals, the fabrication of halogenated anilines from the corresponding nitroarenes was not so straightforward because dehalogenation was usually emerged in the reaction. Thus, chlorinated, brominated, and fluorinated nitroarenes were used to examine the catalytic activity and chemical selectivity of the Fe_3_O_4_@*sn*Pt@PCN. The reaction results demonstrated that the dehalogenation did not occur in this system. All of the halo‐nitroarenes were catalytic transfer hydrogenated to the corresponding anilines in a few minutes (entries 4–9). Then, this Fe_3_O_4_@*sn*Pt@PCN catalyst was also used to the catalytic transfer hydrogenation of nitroarenes with other functional groups, such as, hydroxyl, carboxyl, ester group, ether, amide, amino group, and olefin (entries 10–20). The reaction results indicated that all the functional nitroarenes were successfully transformed to the corresponding anilines with high conversion and selectivity (>99%). It is well known that amino‐substituted heterocycles and diphenyl are significant intermediates for the synthesis of pharmaceuticals and agrochemicals, such as the famous imatinib, paracetamol, hexaflumuron, and the like. Thus, we further applied this catalyst for the catalytic hydrogenation of the nitroarenes which contains heterocyclic ring moieties or other complex group (entries 21–23). The reaction results indicated that all the catalytic hydrogenations proceeded smoothly in few minutes and the substrates were converted into the corresponding heteroaromatic amines with high yields.

**Table 2:**
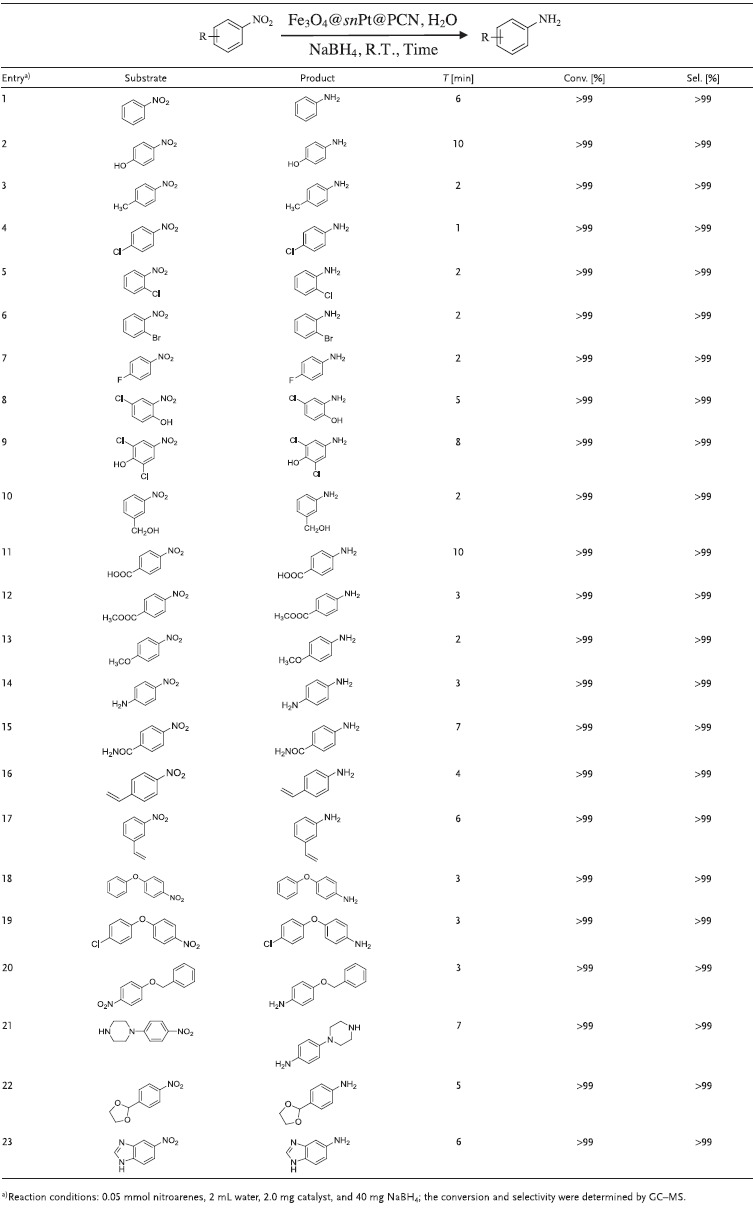
Catalytic transfer hydrogenation of different nitroarenes with NaBH_4_ over the Fe_3_O_4_@*sn*Pt@PCN nanocatalyst

## 3 Conclusion

In summary, we have fabricated an interesting magnetically hollow platinum nanocage and it displays high activity when used as a catalyst nanoreactor. The magnetic hollow nanobox was prepared through the template preparation deposition, oxidative etching, and out shell protection procedures. The thickness of the walls was about subnanometer which contains about six platinum atoms. Due to the high atom utilization efficiency and rapid electronic transform during the reaction, this highly integrated nanomaterial demonstrated high effectiveness for the catalytic transfer of the nitroaromatics to the corresponding anilines. Following reaction, the catalyst can be easily recovered and reused for more than eight times.

## 4 Experimental Section

*Fabrication of Magnetic Hollow Subnano Thick Walls Nanocages—Preparation of Pd Template Nanocubes*: The protocol for the fabrication of Pd nanocubes was the same as previous literature reported.^4^ In a typical procedure, 8 mL of deionized (DI) water was first added to the round‐bottomed flask. Successively, 105 mg of polyvinylpyrrolidone (PVP), 60 mg of ascorbic acid, and 600 mg of KBr were added to the flask. The mixture solution was ultrasonicated for 10 min to dissolve all of the substances. Then, this blend solution was moved to an oil bath and heated at 80 °C for 10 min under magnetic stirring. Subsequently, 3 mL Na_2_PdCl_4_ (20 mg mL^−1^) aqueous solution was added into the preheated solution quickly. The reaction solution was refluxed at 80 °C for another 3 h. The final product was washed with DI water for three times and collected by centrifugation. The precipitate (1.68 mg mL^−1^) was redispersed in 11 mL of ethylene glycol and refrigerated at 4 °C for further use.

*Fabrication of Magnetic Hollow Subnano Thick Walls Nanocages—Preparation of the Pd Nanocubes Supported Nanothick Walls Pt Nanocage (Pd@snPt)*: In a standard synthesis procedure,^4^ 12 mL of ethylene glycol (EG) was first added to the round‐bottomed flask, then 100 mg of ascorbic acid, 54 mg of KBr, and 66.6 mg of PVP were added in succession and this blend solution was ultrasonicated for 10 min. After the substances were completely dissolved, 1.0 mL Pd nanocubes EG solution was added and ultrasonicated for 5 min. The finally mixed solution was heated in an oil bath at 110 °C under magnetic stirring. One hour later, the temperature of the oil bath was quickly ramped to 200 °C with a heating rate of 5 °C min^−1^. Then, 12 mL Na_2_PtCl_6_ EG solution (0.25 mg mL^−1^) was slowly injected into the reaction solution drop by drop at a rate of 4.0 mL h^−1^ via a syringe pipette. After 3 h and all the precursor solution was completely added, the boiling reaction mixture was kept at 200 °C and refluxed for another 1 h. Then, the reaction was cooled to room temperature, and the reaction solution was washed with large amount of ethanol and ultrapure water and collected by centrifugation; finally, the products were redispersed in 1 mL of water.

*Fabrication of Magnetic Hollow Subnano Thick Walls Nanocages—Synthesis of Polydopamine Encapsulated Pd@snPt Core‐Shelled Structure (Pd@snPt@PDA)*: The standard procedure for the synthesis of PDA encapsulated Pd@*sn*Pt core–shell structure was described as the follows: 1 mL of Pd@*sn*Pt nanocubes was first added into 10 mL of tris‐buffer solution (10 × 10^−3^ m) with magnetic stirring at room temperature. Then, 4 mg of dopamine hydrochloride was added into the solution and constantly stirred for 3 h. The resultant product was collected by centrifugation and washed with DI water and ethanol for three times, respectively. The final products were redispersed in 1 mL of water.

*Fabrication of Magnetic Hollow Subnano Thick Walls Nanocages—Synthesis of Hollow Subnano Pt Nanocage Encapsulated by Polydopamine Yolk‐Shelled Nanocubes (snPt@PDA)*: The synthesis of PDA encapsulated hollow subnano Pt nanocage yolk‐shelled nanocubes was achieved via the oxidative etching processing. The Pd template at the center site was etched away by the FeCl_3_ and HCl solution. The detailed procedure was the following: 7 mL of DI water was first added to a round‐bottomed flask, then serially mixed 300 mg of KBr, 50 mg of PVP, 30 mg of FeCl_3_, and 0.18 mL of HCl (pH = 0.55) into this glass reactor. After all substances were dissolved, the mixture solution was put into an oil bath and heated to 100 °C under magnetic stirring. Subsequently, 0.2 mL Pd@*sn*Pt @PDA aqueous solution was introduced into the glass vial by using a syringe pipette. After reacted for 3 h, the product was collected by centrifugation, washed twice with ethanol, and three times with water. The precipitate residue was dried at 60 °C overnight under vacuum.

*Fabrication of Magnetic Hollow Subnano Thick Walls Nanocages—Synthesis of Magnetic Hollow Subnano Pt Nanocage Encapsulated by Carbon (Fe_3_O_4_@snPt@PCN Yolk‐Shelled Nanocubes)*: The as‐prepared snPt @PDA core‐shelled nanocubes were annealed at 500 °C for 3 h in the high purity N_2_ atmosphere with a heating rate of 5 °C min^−1^ to transform into the Fe_3_O_4_@*sn*Pt@PCN yolk‐shelled nanocubes.

*General Procedure for the Catalytic Hydrogen Transfer Reduction of 4‐Nitrophenol by NaBH_4_*: In a typical reaction protocol, 0.1 mmol (13.9 mg) of 4‐nitrophenol and 2.0 mg of Fe_3_O_4_@*sn*Pt@PCN were dispersed into 2 mL distilled water via sonication, first. Then, 40 mg NaBH_4_ was added into the mixture solution at room temperature. The reaction was timely monitored by the UV–vis spectrophotometer and TLC. The final products were analyzed by GC‐MS. Afterward, the catalysts were separated by an extra magnet and reused for the next application.

## Conflict of Interest

The authors declare no conflict of interest.

## Supporting information

Supplementary
