# Supplementary material for: Magnetically Hollow Pt Nanocages with Ultrathin Walls as a Highly Integrated Nanoreactor for Catalytic Transfer Hydrogenation Reaction
Source: Adv Sci (Weinh). 2019 Feb 7;6(7):1802132. doi: 10.1002/advs.201802132 (PMC6446610; doi:10.1002/advs.201802132)
Supplement: Supplementary file 1 — Supplementary [file ADVS-6-1802132-s001.pdf]

Copyright WILEY-VCH Verlag GmbH & Co. KGaA, 69469 Weinheim, Germany, 2019.

ADVANCED SCIENCE
Open Access

Supporting Information

for *Adv. Sci.,* DOI: 10.1002/advs.201802132

Magnetically Hollow Pt Nanocages with Ultrathin Walls as a Highly Integrated Nanoreactor for Catalytic Transfer Hydrogenation Reaction

Yongjian Ai, Zenan Hu, Lei Liu, Junjie Zhou, Yang Long, Jifan Li, Mingyu Ding, Hong-Bin Sun, and Qionglin Liang**

Copyright WILEY-VCH Verlag GmbH & Co. KGaA, 69469 Weinheim, Germany, 2016.

Supporting Information

Magnetically Hollow Pt Nanocages With Ultra-thin Walls as a Highly Integrated Nanoreactor for Catalytic Transfer Hydrogenation Reaction

Yongjian Ai, Zenan Hu, Lei Liu, Junjie Zhou, Yang Long, Jifan Li, Mingyu Ding, Hong-bin Sun,* and Qionglin Liang*

Table of content

1. General information of characterization..S2

2. Materials characterization...S3-S8

3. GC-MS Data of Anilines……………………………......................................S9-S13

Referances

1. General information of characterization

The morphology of the catalyst was characterized by the High Resolution Transmission Electron Microscopy (HR-TEM; JEOL JEM-2100F field emission electron microscope) which was operated with 200 kV at room temperature (25 °C). Scanning transmission electron microscopy (STEM) and Energy Dispersive Spectrometer (EDS) analyzer are the accessory instruments of the HR-TEM apparatus. HAADF-STEM and EDS analyses were performed using an Filed Emission aberration-corrected microscope STEM/TEM (FEI Titan 80-300). The catalysts' X-ray diffraction (XRD) experiments were recorded on a rigaku corporation Smart Lab, diffractometer using Cu Kα radiation (λ = 0.1541 nm). X-ray photoelectron spectroscopy (XPS) measurements were performed by using a PHI Quantera SXM spectrometer, ULVAC-PHI, the binding energy determination was based on the carbon contamination C 1s at 284.8 eV with an experimental error of ±0.2 eV. The solvent of TLC was petroleum ether and ethyl acetate mixed solution, the UV-Vis spectroscopy was 434003-UV-Vis Analyst and the GC-MS was used Bruker 450GC-320MS.

2. Materials characterization

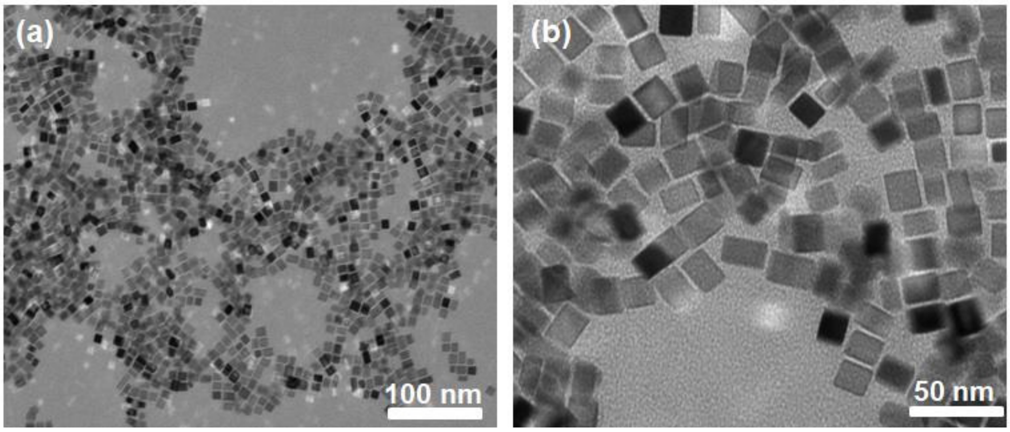

Figure S1. HR-TEM images of Pd nanocube.

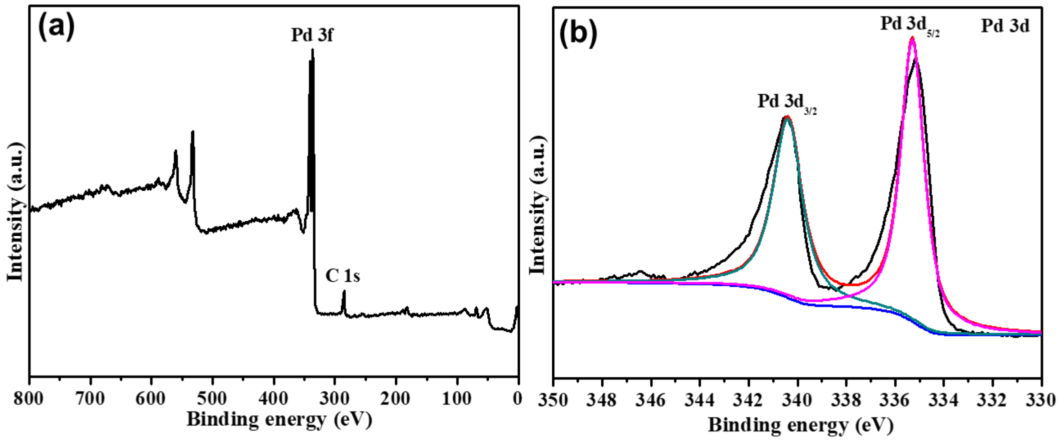

Figure S2. XPS characterization of the Pd nanocubes (a) survey spectra, (b) Pd 3d spectra.

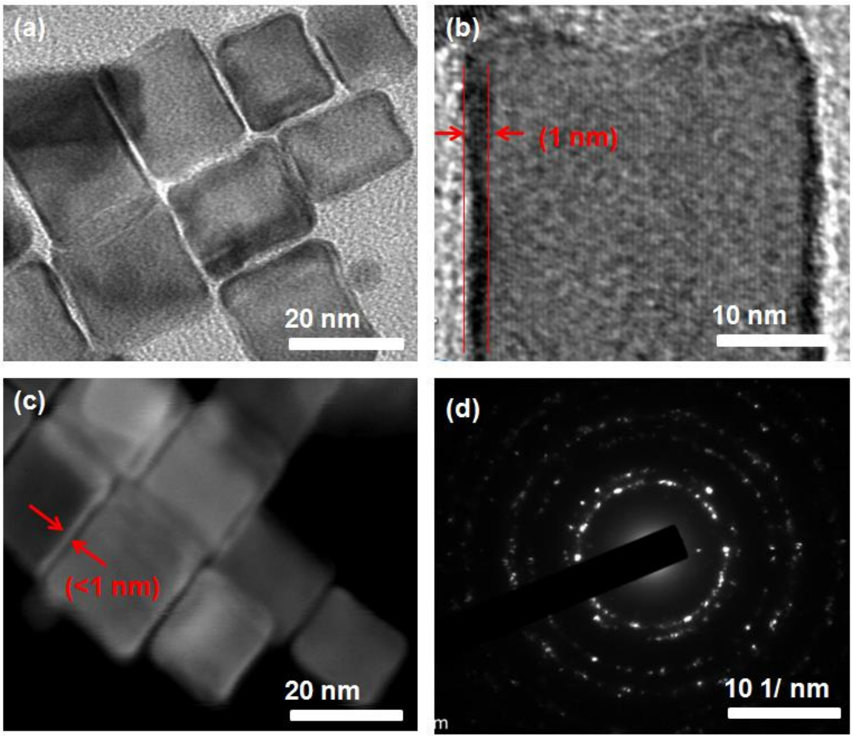

Figure S3. (a-b) HR-TEM, (c) STEM, (d) SAED images of Pd@snPt.

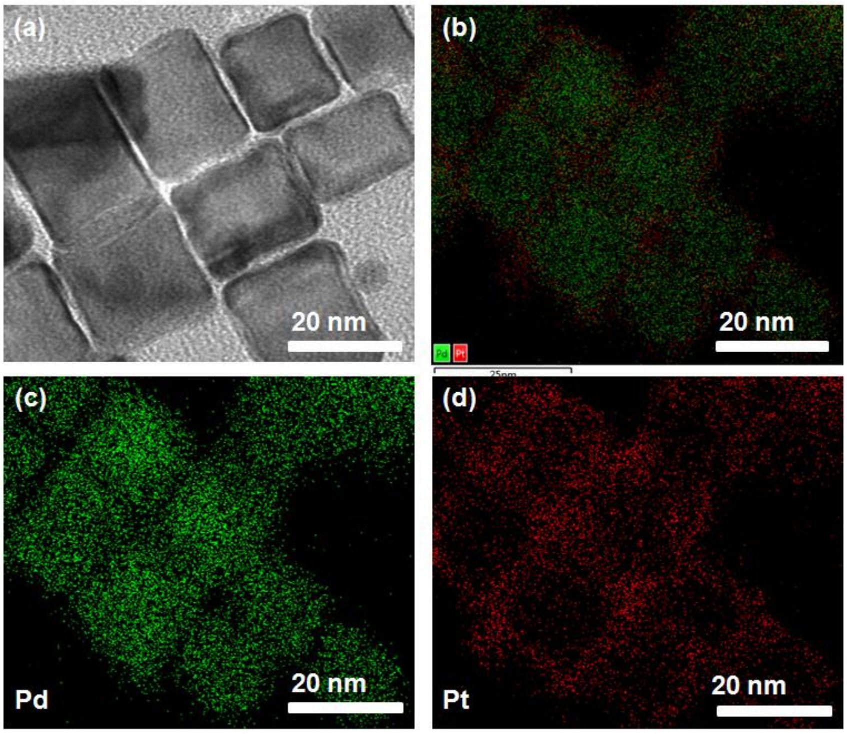

Figure S4. (a-b) HR-TEM images of Pd@snPt; (b) EDS mapping of Pd@snPt, (c) Pd (d) Pt in Pd@snPt.

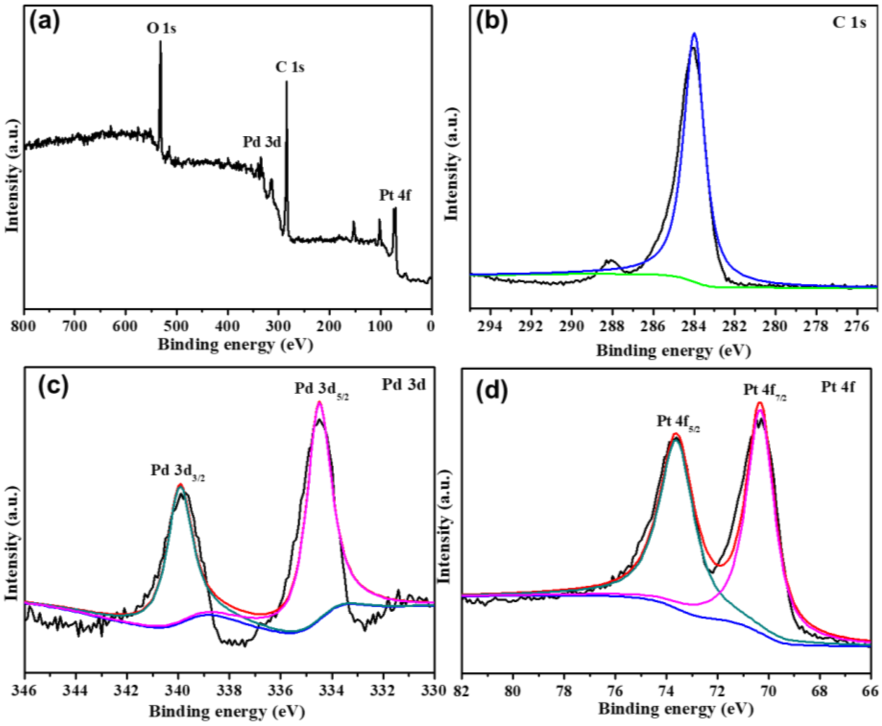

Figure S5. XPS characterization of the Pd@snPt (a) survey spectra, (b) C 1s spectra, (c) Pd 3d spectra, (d) Pt 4f spectra.

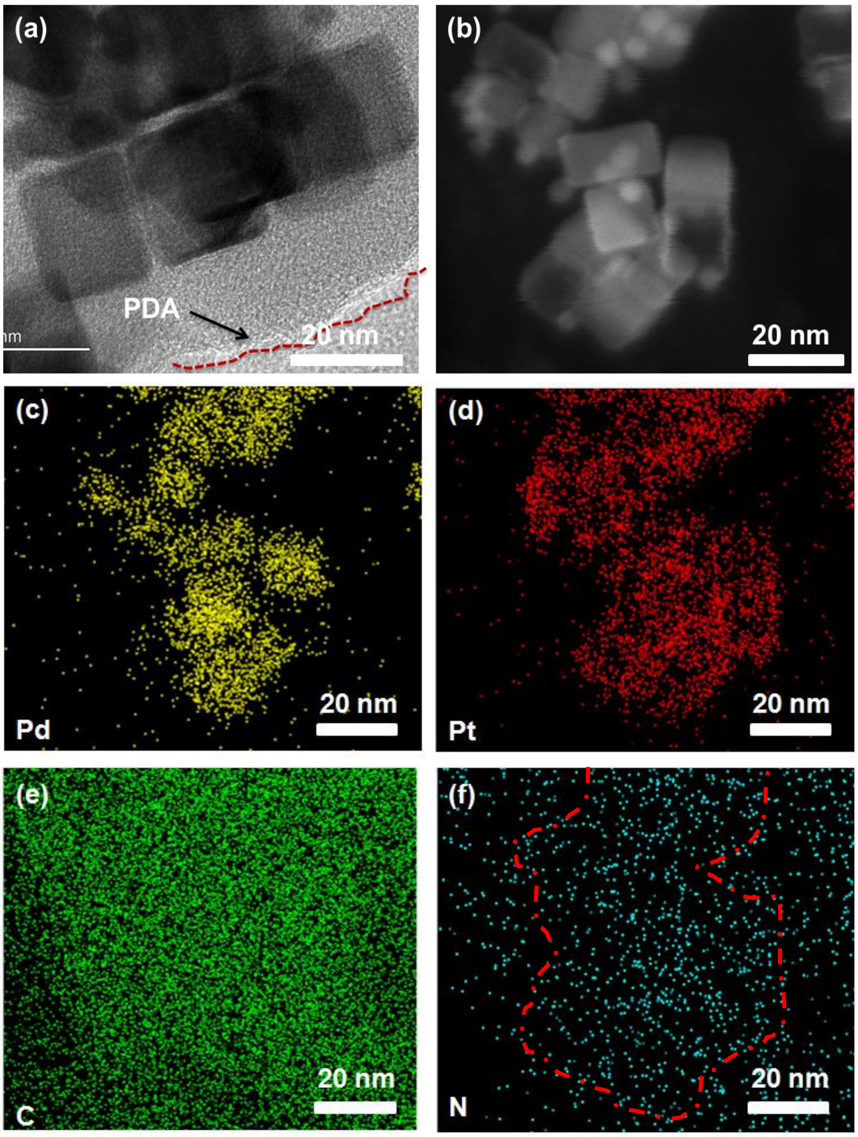

Figure S6. (a) HR-TEM, (b) STEM images of Pd@snPt@PDA, (c) EDS mapping of Pd, (d) Pt, (e) C, (f) N in Pd@snPt@PDA.

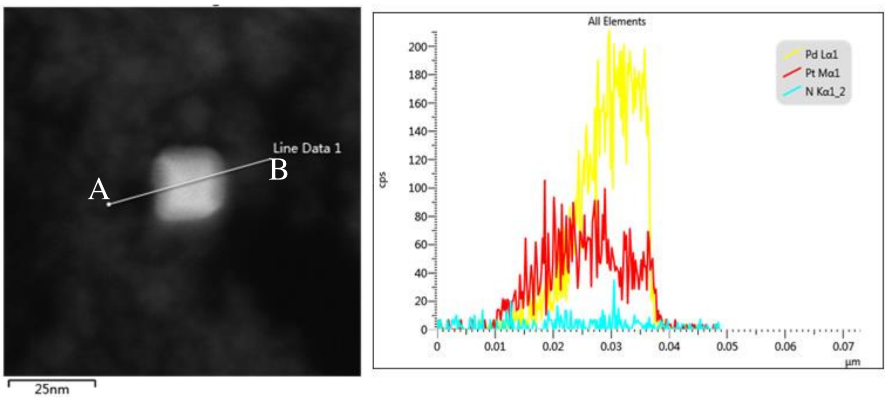

Figure S7. (a) STEM images of Pd@snPt@PDA; (b)EDS elemental line scan from A to B showing the presence of Pd, Pt and N elements.

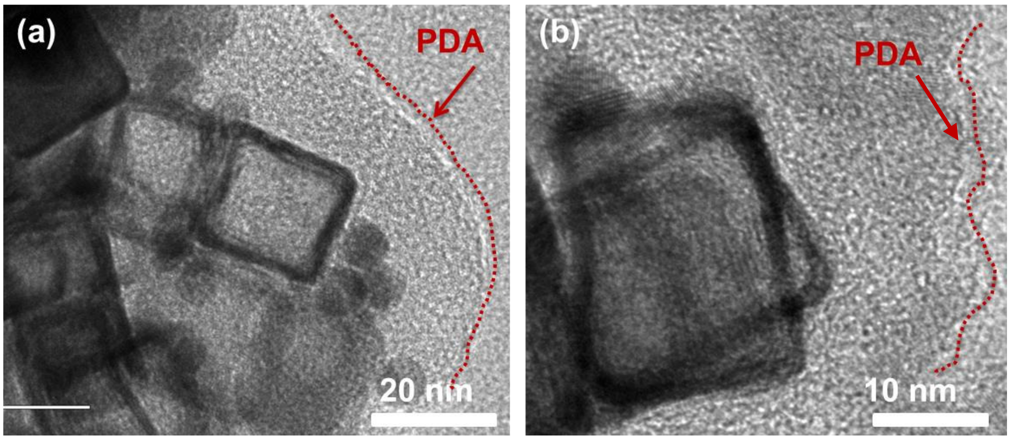

Figure S8. HR-TEM images of snPt@PDA.

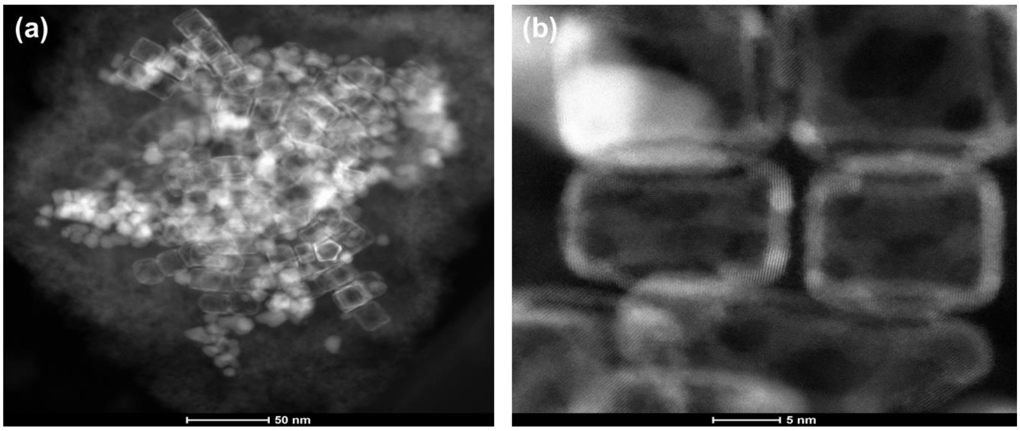

Figure S9. HAADF-STEM images of the Fe_3O_4@snPt@PCN nanocatalyst.

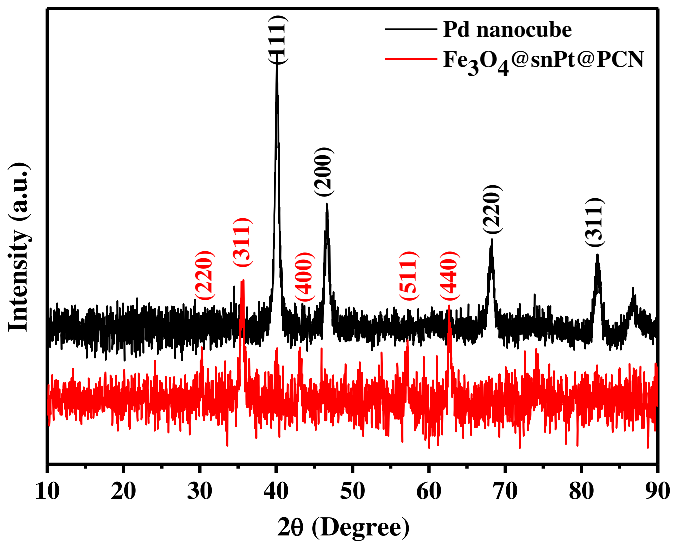

Figure S10. XRD pattern of Pd nanotube and Fe_3O_4@snPt@PCN.

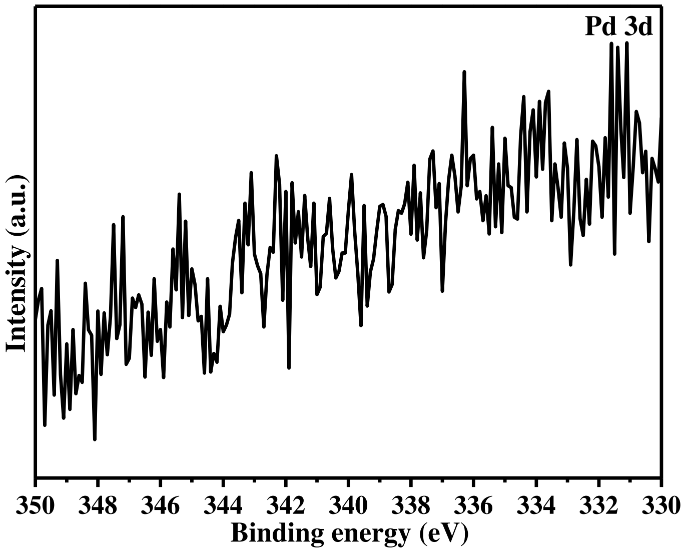

Figure S11. XPS spectra of the Pd after oxidation etching treatment.

`

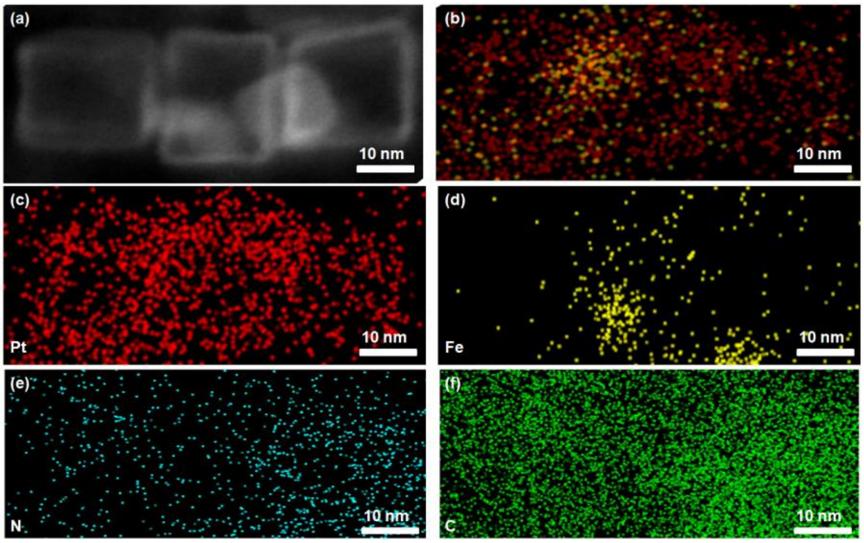

Figure S12. (a) STEM images and (b) ESD mapping of the Fe_3O_4@snPt@PCN after reused for 8 times.

3. GC-MS Data of Anilines:

All compounds were identified by GC-MS and comparison with fragmentation pattern. GC-MS data of isolated compounds is compared with literature value and given here[1-6].

Anilines (Table 2, entry 1)

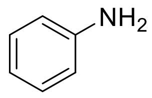

GC-MS: m/z (%) 93 (100) $[M]^+$, 66 (34), 39 (14).

4-Aminophenol (Table 2, entry 2):

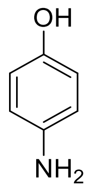

GC-MS: m/z (%) 109 (100) $[M]^+$, 80 (50), 52 (14).

4-Toluidine (Table 2, entry 3):

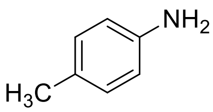

GC-MS: m/z (%) 106 (100) $[M]^+$, 77 (12).

4-Chlorobenzenamine (Table 2, entry 4):

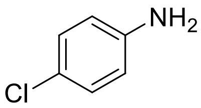

GC-MS: m/z (%) 127 (100) $[M]^+$, 92 (13), 65 (21).

2-Chloroaniline (Table 2, entry 5):

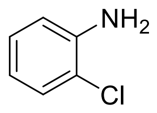

GC-MS: m/z (%) 127 (100) $[M]^+$, 92 (14), 65 (17).

3-Bromoanilines (Table 2, entry 6)

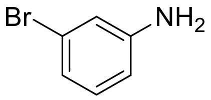

GC-MS: m/z (%) 173 (100) $[M]^+$, 92 (91), 65 (85).

4-Fluoroaniline (Table 2, entry 7)

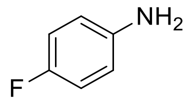

GC-MS: m/z (%) 111 (100) $[M]^+$, 84 (43), 57 (11).

2-Hydroxy-5-chloro-aniline (Table 2, entry 8):

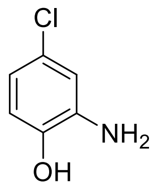

GC-MS: m/z (%) 143 (100) $[M]^+$, 114 (14), 80 (31), 51 (10).

2,6-Dichloro-4-aminophenol (Table 2, entry 9):

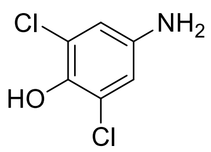

GC-MS: m/z (%) 177 (100) [M]+, 113 (87), 78 (60), 52 (16).

(3-Aminophenyl)-methanol (Table 2, entry 10):

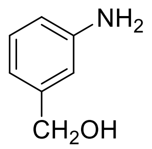

GC-MS: m/z (%) 123 (100) $[M]^+$, 94 (74), 77 (30), 65 (13), 39 (8).

4-Acetylaminobenzoic acid (Table 3, entry 11):

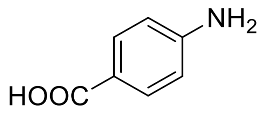

GC-MS: m/z (%) 120 (100) $[M]^+$, 92 (32), 65 (26), 39 (8).

Methyl 4-aminobenzoate (Table 2, entry 12):

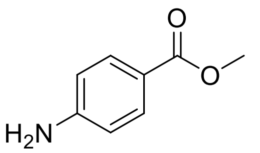

GC-MS: m/z (%) 151 (54) $[M]^+$, 120 (100), 92 (26), 65 (20).

4-Methoxyaniline (Table 2, entry 13):

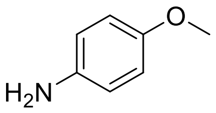

GC-MS: m/z (%) 108 (100) $[M]^+$, 80 (36), 53 (14).

1,4-Benzenediamine (Table 2, entry 14):

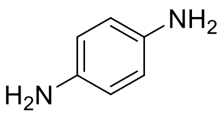

GC-MS: m/z (%) 108 (100) $[M]^+$, 80 (33), 53 (10).

4-Aminobenzamide (Table 2, entry 15):

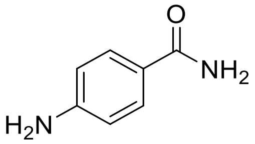

GC-MS: m/z (%) 136 (73) $[M]^+$, 120 (100), 92 (34), 65 (27), 39 (8).

4-Aminostyrene (Table 2, entry 16):

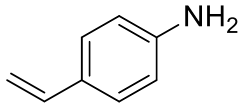

GC-MS: m/z (%) 119(100) $[M]^+$, 91 (23), 65 (18), 39 (13).

3-Aminostyrene (Table 2, entry 17):

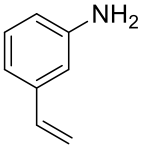

GC-MS: m/z (%) 119 (100) [M]+, 91 (28), 89 (5), 65 (13).

4-Aminodiphenyl ether (Table 2, entry 18):

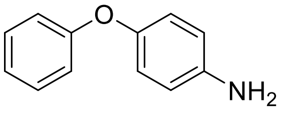

GC-MS: m/z (%) 185 (100) $[M]^+$, 156 (17), 108 (74), 80 (25), 51 (13).

4-(4-Chlorophenoxy)benzenamine (Table 2, entry 19):

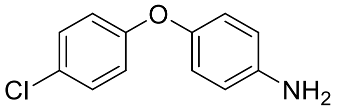

GC-MS: m/z (%) 219 (72) $[M]^+$, 156 (13), 108 (100), 80 (30).

4-(Benzyloxy)benzenamine (Table 2, entry 20):

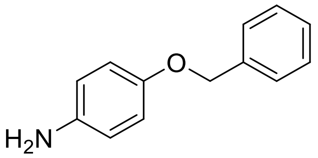

GC-MS: m/z (%) 199 (15) $[M]^+$, 108 (100), 91 (29), 80 (14), 53 (5).

1-(4-Aminophenyl)piperazine (Table 2, entry 21):

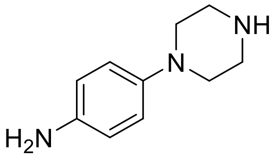

GC-MS: m/z (%) 177 (51) $[M]^+$, 135 (100), 120 (30), 92 (10) 65 (11).

5-(Aminophenyl)-1,3-dioxolane (Table 2, entry 22):

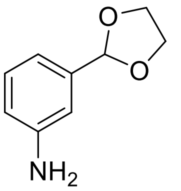

GC-MS: m/z (%) 165 (99) $[M]^+$, 120 (52), 93 (100), 65 (26).

6-Amino-1H-benzimidazole (Table 2, entry 23):

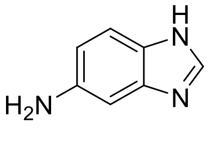

GC-MS: m/z (%) 133 (100) $[M]^+$, 106 (15), 78 (8), 52 (12).

Referances

[1]. R. J. Rahaim, R. E. Maleczka, Org. Lett. **2005**, 7, 5087.

[2]. AIST: Integrated Spectral Database System of Organic Compounds. (Data were obtained from the National Institute of Advanced Industrial Science and Technology (Japan)).

[3]. U. Sharma, P. K. Verma, N. Kumar, V. Kumar, M. Bala, B. Singh, Chem. Eur. J. **2011**, 17, 5903.

[4]. J. Zhang, G. Lu, C. Cai, Catal. Commun. **2016**, 84, 25.

[5]. J. Zhou, Y. Li, H. Sun, Z. Tang, L. Qi, L. Liu, Y. Ai, S. Li, Z. Shao, Q. Liang, Green. Chem. **2017**, 19, 3400.

[6]. Y. Ai, Z. Hu, Z. Shao, L. Qi, L. Liu, J. Zhou, H. Sun, Q. Liang, Nano. Res. **2018**, 11, 287.
